# Blended and digital approaches in histology and pathology teaching: A scoping review

**DOI:** 10.1002/ase.70169

**Published:** 2025-12-26

**Authors:** Eleonora Nava, Ashis Jalote‐Parmar, Cecilie Våpenstad, Marit Valla

**Affiliations:** ^1^ Faculty of Medicine and Health Sciences, Department of Clinical and Molecular Medicine (IKOM) NTNU Norwegian University of Science and Technology Trondheim Norway; ^2^ Clinic of Laboratory Medicine St. Olav's Hospital, Trondheim University Hospital Trondheim Norway; ^3^ Faculty of Architecture and Design, Department of Design NTNU Norwegian University of Science and Technology Trondheim Norway; ^4^ Department of Health Research SINTEF Digital, SINTEF Trondheim Norway

**Keywords:** blended learning, digital learning, digital microscopy, histology, medical education, pathology

## Abstract

Histology and pathology education is evolving, driven by the integration of digital microscopy with other technological advances. Gaining insight into the impact of this transition, while understanding the perspectives of both students and educators, is important for improving teaching practices. This includes mapping teaching methods, digital learning tools and assessment strategies in digital or blended learning environments, and exploring interactions between students and educators. This scoping review investigated how the integration of digital microscopy into histology and pathology education, and a shift to digital and blended learning, has impacted teaching and learning practices. Five databases were used in the search (PubMed, EMBASE, ERIC ProQuest, Web of Science, and Scopus), resulting in 98 included articles. The literature indicated that teaching methods have become more accessible, interactive, and student‐centered than traditional instruction. Thirty studies described digital learning tools with digital microscopy, which were used by educators for teaching and by students for independent studies. Image‐based quizzes and formative feedback for self‐evaluation were frequently reported assessment methods. The lack of physical interaction between students, peers and educators was reported to be a disadvantage of digital learning. Several studies identified blended approaches as a possible solution to maintain accessible digital teaching while preserving valuable student‐educator interactions. This review discusses the findings within a broader perspective, identifies opportunities to enhance the learning experience for educators and medical students, and addresses diverse needs to guide the future development of digital learning tools.

## INTRODUCTION

In recent years, the integration of digital microscopy into medical education has gained significant attention, driven by the need to modernize teaching methods^1^, ^2^ and to handle an increasing number of medical students.^3^, ^4^ Studies have shown that digital microscopy has similar learning outcomes compared with traditional microscopy courses^2^ and is shown to provide a more flexible and interactive learning environment.^5^ In this article, the term digital microscopy refers to the use of digitized tissue slides and associated viewing software, often referred to in the literature as virtual microscopy. This framing is adopted for consistent terminology across the text and to ensure conceptual clarity.

Histology and pathology are fundamental components of medical education, providing students with essential knowledge about the structural and functional organization of healthy and pathological tissues and organs at the cellular level. In histology, students learn what normal tissue looks like in a microscope. In pathology, students learn about disease processes and the morphologic changes that take place in pathological tissue.^6^ Histology and pathology teaching is traditionally rooted in microscope‐based learning involving the use of physical light microscopes to examine tissue slides.^7^, ^8^ During microscopy courses, educators guide students in assessing slides, pointing out important structures and features, thus helping students develop visual understanding and pattern recognition skills.^1^, ^9^, ^10^

With the introduction of digital microscopy, physical glass slides were scanned and can be examined on a computer screen or other digital devices, instead of under a microscope.^11^ The digitized images, or whole slide images (WSI), can also be viewed, annotated, and shared using digital microscopy software. This has opened new possibilities for teaching,^8^, ^12^ modernizing teaching methods and tools used.^1^, ^2^ Digital microscopy also allows educators to show the same section to all students simultaneously, allowing them to interact with it at the same time on their own screen.^13^

Digital microscopy has made microscopy more accessible to students,^13^ allowing them to practice anytime and anywhere. It may improve learning outcomes,^14^ and enable more dynamic and engaging teaching experiences.^15^ During the COVID‐19 pandemic, most universities and institutions were forced to switch from traditional to digital microscopy quickly, and digital microscopy played a critical role in maintaining continuity in medical education.^16^, ^17^ Post‐pandemic, digital tools have been integrated into histology and pathology teaching, and a redesign of microscopy courses has occurred globally.^18^, ^19^

The use of digital microscopy and digital tools has been found to improve histology and pathology microscopy training, providing a more interactive and active learning experience.^20^, ^21^ Educators can engage students through activation, and use interactive quizzes or polls to give them instant feedback and reinforce their learning.^16^ Students have been encouraged to work in groups to solve problems or discuss cases and to study more on their own.^22^ Traditional teaching tools, such as textbooks, printed atlases, and physical handouts can complement online tutorials, digital atlases, recorded video lectures and discussion boards on social media.^1^

Establishing and maintaining digitized slide collections or implementing digital microscopy software or platforms is, however, costly.^23^ Digital microscopy teaching may require rich tissue slide collections and investment in high‐resolution scanners, digital storage solutions, and secure, accessible platforms.^24^ An alternative to establishing new digital slide collections is to use digital microscopy repositories, such as the Virtual Microscopy Database,^25^ where digitized slides can be uploaded, shared and downloaded among institutions. High‐resolution scanning is necessary to capture morphological details in the tissue slides, resulting in extremely large digital files.^26^ The large file size of digital tissue slides makes it necessary to use specialized software for viewing and analyzing the images.

Prior literature that mapped the evolution of histology and pathology teaching recognized the importance of digital microscopy in future education,^1^, ^12^, ^22^, ^27^ highlighted its advantages^28^ and challenges,^24^ and offered an overview of online educational resources available during the COVID‐19 pandemic.^17^ Despite this, there is still limited understanding of how digital microscopy impacts students' learning experiences, and how digital learning tools with digital microscopy integrated are being used in histology and pathology education. Understanding how digital microscopy can be effectively integrated into digital learning tools, and into education more broadly, remains crucial. Moreover, more insights into assessment methods and how students and educators interact in a digital context are needed.

### Study purpose

The objective of this scoping review was to examine current digital and blended teaching methods, digital learning tools, digital assessment methods, interactions between students and educators, and their perceptions of the transition from traditional to digital or blended learning in histology and pathology education.

## METHODS

The scoping review was conducted in accordance with the JBI methodology,^29^, ^30^ ASE guidelines,^31^ and PRISMA‐ScR protocol.^32^ A scoping review method was chosen due to its exploratory nature, allowing for an examination of the scope, breadth, and depth of the literature.^33^, ^34^ It was found to be the most appropriate method to address the study's research question and to identify and provide an overview of the ongoing integration of digital microscopy in histology and pathology education.

### Identification of relevant studies

Relevant search terms were identified through a collaborative approach among researchers from different fields of expertise: histology and pathology education (M.V.), medical technology and science and technology studies (C.V.), and interaction design and technology (A.J.P and E.N.). An electronic search of scientific literature was conducted using the following five databases: PubMed (US National Library of Medicine, National Institutes of Health, Bethesda, MD), EMBASE (Ovid Technologies, Inc., New York, NY), ERIC (ProQuest, Ann Arbor, MI), Web of Science (Clarivate Analytics, Philadelphia, PA) and Scopus (Elsevier B.V., Amsterdam, Netherlands). A research librarian at NTNU assisted the authors in identifying relevant databases and refining the search strategy. A pilot search using PubMed, EMBASE and Web of Science was conducted prior to the systematic search to identify relevant articles and search terms associated with those.

The search string (see Appendix A) covered two main concepts. The first concept was related to histology and pathology, and the terms searched were histology, pathology, “microscopic anatomy,” histopathology, and “virtual microscopy.” The second concept limited the results to digital learning and the terms searched were “digital learning,” “online learning,” “e‐learning,” “hybrid learning,” and “blended learning.” Synonyms were used for both concepts, with “Histology” in PubMed and Embase and “Pathology” in PubMed, Embase, Web of Science and Eric ProQuest for the first concept, and “Education, Distance” in PubMed, “Distance learning” in Embase, “Distance Education” and “Electronic Learning” in Eric ProQuest for the second concept.

The first author performed the systematic search on May 24, 2024, and updated the search on September 1, 2024. Techniques for searching included the use of search tools such as Boolean operators, synonyms, and free text terms. The search was customized for each selected database in accordance with their filtering specifications. In all five databases, the search was limited to studies published in English from January 2013 to September 2024. The timeline was selected to identify articles that reflected recent changes in histology and pathology education. In Web of Science and Embase, the search was filtered to original studies, while for the other databases, a manual selection was performed to identify original research.

### Eligibility criteria

The eligibility criteria were developed using the Population, Concept, and Context (PCC) framework.^30^ Population defined the groups of interest in the study, the Concept stated the main topic, and the Context indicated the setting or environment.

*Population*: Studies about medical students and educators were included. Studies regarding healthcare professionals working in diagnostic pathology were excluded.

*Concept*: Studies describing teaching methods related to histology and pathology education in a blended or digital learning environment, digital learning tools, assessment methods, interaction between students and educators, and perspectives on traditional versus digital or blended learning were included. Studies that were not related to teaching were excluded.

*Context*: Studies focusing on microscopy teaching in histology and pathology were included. Studies on macroscopic histology and pathology were excluded.

*Study characteristics*: Included criteria were studies categorized as original research including experimental, observational (both analytical and descriptive), and qualitative designs. Exclusion criteria were studies published before 2013, articles not written in English, non‐original research (such as reports, conference abstracts, editorial letters, commentaries, all types of reviews and theses).

### Study screening and selection

**FIGURE 1:**
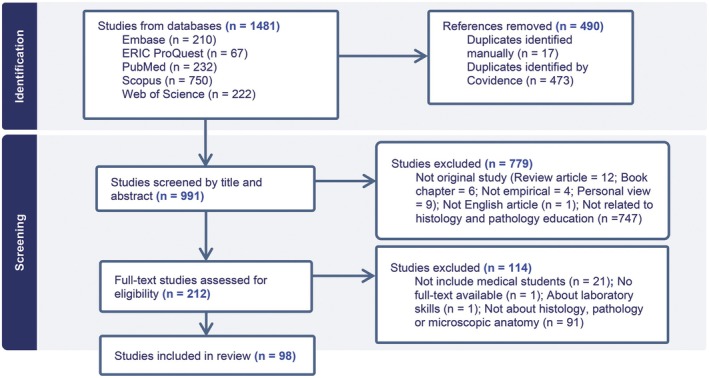
PRISMA flow diagram of article selection. Out of 1481 articles identified, 490 duplicates and 893 articles were removed throughout the selection process.

To facilitate the review process, Covidence software (Veritas Health Innovation, Melbourne, Australia) was used. The screening process involved all four authors and included automatic and manual duplicate record removal. In the first stage of screening by title and abstract, each article was screened by two authors. The first author (EN) screened ~50% of the articles, with the remaining articles divided among the other three authors. Discrepancies between authors were discussed and resolved by consensus in meetings where all authors participated. Inter‐rater agreement was assessed using Cohen's Kappa and interpreted as follows: 0.00–0.20 = “slight,” 0.21–0.40 = “fair,” 0.41–0.60 = “moderate,” 0.61–0.80 = “substantial,” and 0.81–1.00 = “almost perfect”.^35^ The inter‐rater agreement ranged from 0.44 to 0.74, suggesting moderate to substantial agreement between the authors. In the full‐text review, each article was analyzed according to preset criteria and was analyzed by one author. The first author (EN) screened half of the articles, and the remaining half were divided among the other three authors. All authors met to resolve any uncertainties about the eligibility of the articles after full‐text review. Figure 1 reports the flow of studies through the different stages of the screening process in a PRISMA flow diagram.

### Data extraction

The data extraction template was tailored to meet the specific needs of the review while remaining aligned with the guidance from the Cochrane Qualitative and Implementation Group.^36^ Information charted included the year of publication, first author's surname, country of the authors' affiliations, study design, participant educational level (1st year, 2nd year…), number of participants (if specified), and if related to histology or pathology. Extracted data covered teaching methods, digital learning tools (including type, name, data collection methods, and brief outcome descriptions, if available), assessment methods, student–educator interactions, and traditional versus digital or blended learning (including data collection methods for perspectives). A pilot test of the data extraction template was conducted prior to implementation. Any disagreements during data extraction were resolved through discussion and consensus.

### Collating, summarizing, and reporting results

The data were extracted from Covidence into Microsoft Excel (Microsoft Corporation, Redmond, WA, USA). Results were organized around five primary themes, and within each theme, data were systematically summarized to capture common practices, variations, and emerging trends. Quantitative data were tabulated to illustrate frequency and distribution, while qualitative data were summarized narratively to offer contextual insights.

## RESULTS

A total of 1481 articles were identified through database searches, as reported in Figure 1 below. After removing 490 duplicates, 991 unique articles were screened for eligibility by reading the title and abstract. In the title and abstract screening, 779 studies were excluded. The remaining 212 full‐text articles were sought for eligibility, and finally, 98 studies were included.

### Characteristics of the included studies

**FIGURE 2:**
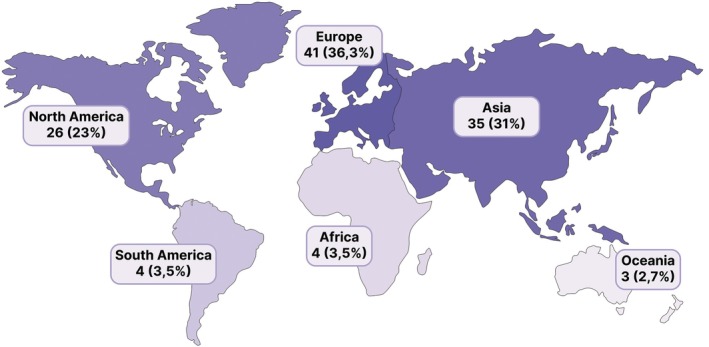
Geographical distribution of the included studies across six continents based on authors' affiliations, highlighting Europe, Asia, and North America as the main contributing regions.

The geographical distribution of affiliated authors in the included articles was as follows (Figure 2): Europe 41 studies (36.3%), Asia 35 studies (31%), North America 26 studies (23%), South America and Africa four studies each (3.5% each), and Oceania three studies (2.7%). The United States contributed with the highest number of articles (*n* = 24), followed by Germany (*n* = 11), and India (*n* = 10). In studies involving authors from multiple countries, each country was counted.

**FIGURE 3:**
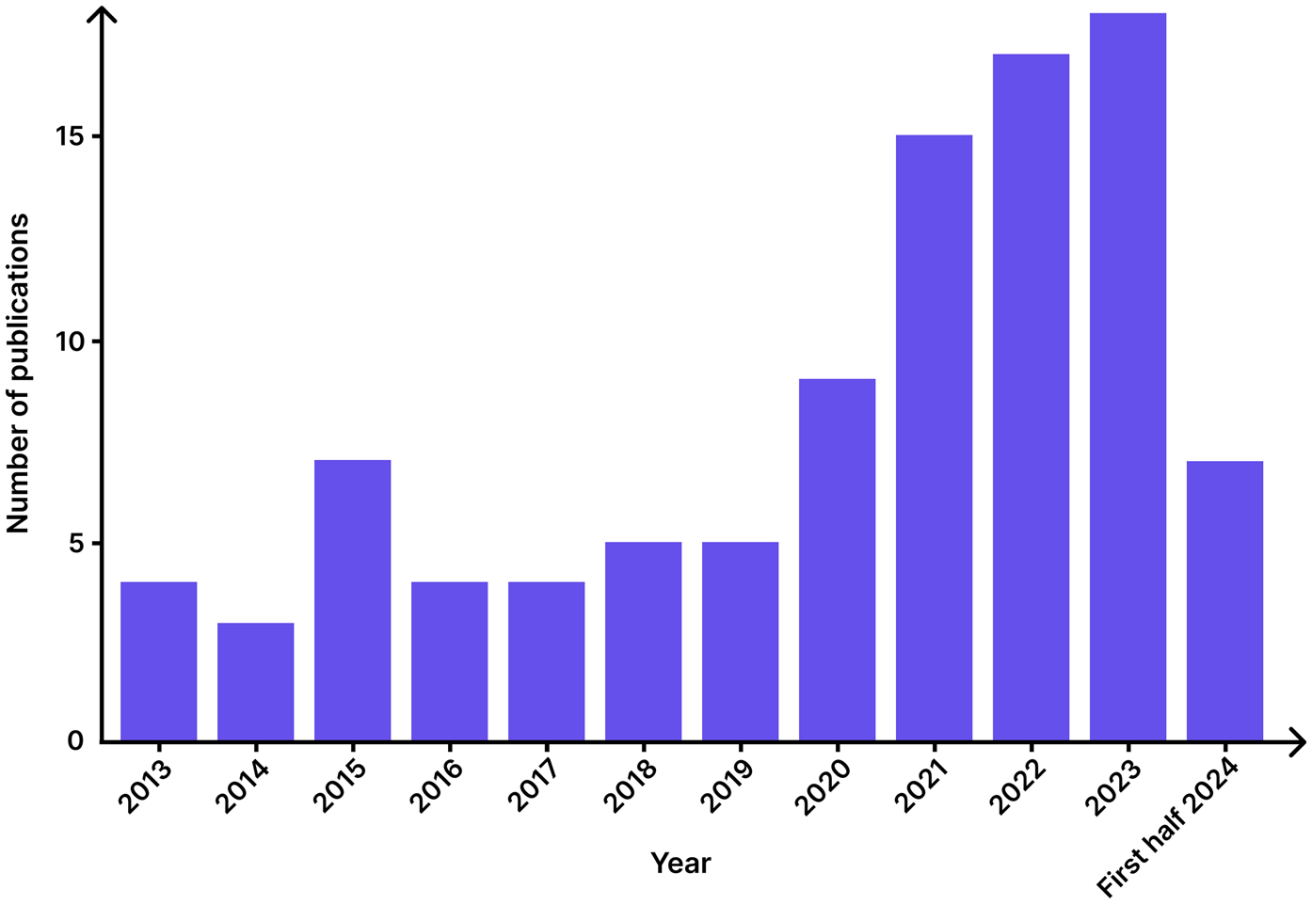
Publication year of the included studies, showing a steady increase in research output from 2013 to the first half of 2024.

From 2013 to 2019, there were five publications per year on average (Figure 3). There were nine publications in 2020. From 2021 to 2023, the number of publications continued to increase, reaching 18 in 2023. In September 2024, when the database search ended, seven studies were identified.

First‐year medical students were the most frequently represented group, appearing in 26 studies, followed by second‐year students in 22 studies. Third‐year students appeared in seven studies, fourth and fifth‐year students in six each, and sixth‐year students in one study. In 30 studies, the students' education level was not specified. Table 1 lists the study design and subjects of the included studies, as categorized by the authors.

**TABLE 1:** Study design and subjects of the included studies mentioned and categorized by the review authors.

Study characteristics		*n* (%)
Study design	Case study	35 (35.8%)
	Cross‐sectional study	15 (15.3%)
	Non‐randomized study	12 (12.2%)
	Cohort study	7 (7.1%)
	Randomized controlled trial	6 (6.1%)
	Mixed methods	2 (2%)
	Not identified	21 (21.5%)
Subjects	Histology	47 (48%)
	Pathology	26 (26.5%)
	Both	25 (25.5%)

### Teaching methods

The theme “teaching methods” explored the instructional approaches and strategies employed by institutions or educators to facilitate histology and pathology learning in a digital or blended environment. Table 2 provides an overview of the different teaching methods identified, including how often each method appeared in the articles. E‐learning modules were the most frequently used method.

**TABLE 2:** Categorization of the teaching methods found in the included articles.

Teaching method	Definition	*n*	References
E‐learning module	Asynchronous, online modules combining video lessons, tutorials, quizzes and interactive activities	48	[27, 28, 37, 38, 39, 40, 41, 42, 43, 44, 45, 46, 47, 48, 49, 50, 51, 52, 53, 54, 55, 56, 57, 58, 59, 60, 61, 62, 63, 64, 65, 66, 67, 68, 69, 70, 71, 72, 73, 74, 75, 76, 77, 78, 79]
Virtual classroom	Synchronous, online lectures	33	[47, 60, 62, 68, 75, 78, 79, 80, 81, 82, 83, 84, 85, 86, 87, 88, 89, 90, 91, 92, 93, 94, 95, 96, 97, 98, 99, 100, 101, 102, 103, 104, 105, 106, 107]
Physical classroom	Synchronous, face‐to‐face lectures	17	[37, 46, 47, 60, 65, 74, 79, 83, 84, 88, 89, 93, 95, 107, 108, 109, 110]
Recorded video lectures	Asynchronous, recorded materials	19	[51, 59, 68, 74, 79, 80, 82, 83, 85, 86, 90, 93, 98, 100, 105, 106, 111, 112]
Collaborative learning	Students discussed topics in groups, presented findings, and gave feedback to peers	16	[44, 51, 53, 55, 57, 62, 70, 71, 86, 87, 90, 96, 110, 113, 114, 115, 116]
Flipped classroom	Independent studies before class, combined with active engagement during lectures	14	[44, 51, 62, 65, 70, 76, 86, 88, 112, 117, 118, 119, 120, 121]
Case‐based learning	Real‐life or simulated cases addressed individually or in groups	13	[27, 54, 55, 57, 62, 64, 68, 76, 86, 88, 99, 100, 114]
Interactive multimedia	Incorporating videos, animations, and social media for dynamic learning experiences	11	[51, 60, 66, 82, 86, 93, 99, 100, 103, 106, 115]
Gamification	Integrating game activities to foster engagement	8	[62, 68, 82, 87, 95, 113, 114, 115]
Problem‐based learning	Focusing on exploratory problem‐solving through cases, either individually or in groups	6	[44, 62, 86, 88, 114, 117]
Team‐based learning	Structured group work	6	[53, 62, 71, 86, 114, 122]
Adaptive learning	Relying on data‐driven algorithms to personalize learning experiences	4	[38, 45, 61, 63]
Mobile learning	Learning activities via mobile devices	4	[47, 72, 109, 122]
Simulation‐based learning	Employing extended reality technologies like Virtual Reality (VR) or Augmented Reality (AR)	2	[86, 103]
Concept mapping	Using diagrams to visualize concepts	1	[115]

### Digital learning tools

The theme “digital learning tools” was used to refer to digital technologies and platforms that supported and delivered histology and pathology educational content in a digital environment. Table 3 provides an overview of the different categories of digital learning tools identified in the included articles. Table 4 lists all digital tools used in digital and blended learning, that did not integrate digital microscopy but still supported static image sharing and use. Table 5 lists digital learning tools with digital microscopy integrated. A large range of digital learning tools has been used in the teaching of histology and pathology, from commonly available tools such as PowerPoint and Zoom to more specialized tools integrating digital microscopy.

**TABLE 3:** Categorization of the digital learning tools found in the included articles.

Digital learning tools	Definition	*n*	References
Web‐based tools with digital microscopy integrated	Interactive digital tools with digitized slides integrated, annotations, quizzes, and additional materials	30	[40, 41, 43, 45, 46, 47, 52, 54, 55, 56, 57, 58, 59, 61, 62, 63, 65, 66, 67, 71, 72, 73, 74, 75, 78, 79, 86, 89, 93, 97]
Video conferencing tools	Video communication platforms (software such as Zoom, Microsoft Teams, Google Meets)	28	[47, 51, 54, 59, 62, 68, 78, 82, 85, 86, 89, 90, 91, 92, 94, 96, 97, 100, 101, 102, 103, 104, 105, 106, 112, 113, 118, 121]
Learning Management Systems (LMS)	Platform for managing and delivering e‐learning (such as Moodle, Canvas, Blackboard)	26	[27, 37, 42, 44, 47, 49, 50, 51, 62, 64, 68, 74, 76, 78, 80, 82, 83, 86, 89, 96, 100, 102, 106, 118, 119, 122]
Digital atlases and repositories of digitized slides	Online collections of high‐resolution and categorized histological/pathological digitized slides	24	[25, 39, 42, 53, 64, 69, 77, 81, 82, 83, 100, 105, 106, 108, 109, 114, 118, 120, 123, 124, 125, 126, 127]
Presentation software	Tools for creating and displaying visual slideshows (such as PowerPoint)	23	[39, 42, 49, 59, 62, 74, 79, 81, 82, 84, 85, 86, 90, 93, 99, 101, 102, 104, 114, 117, 118, 119, 128]
Online interactive modules	Self‐paced learning units with interactive elements (such as MOOC, Digital online courses)	14	[27, 28, 48, 50, 59, 65, 69, 76, 86, 98, 103, 106, 112, 119]
Interactive live quizzes	Real‐time quizzes with immediate feedback (such as Poll Everywhere, Kahoot!)	13	[46, 62, 68, 82, 83, 86, 88, 95, 100, 101, 113, 118, 119]
Discussion boards and social media	Online spaces for communication and idea exchange (such as forum, blog, social media, podcasts)	9	[57, 66, 86, 97, 103, 106, 111, 118, 121]
E‐books and digital textbooks	Digital format of books	7	[59, 68, 87, 91, 93, 100, 115]
Collaborative platforms for group work	Tools for team‐based online project collaboration (such as Google Docs, Miro)	5	[54, 84, 96, 99, 116]

**TABLE 4:** Software and digital learning tools supporting static image sharing, but without digital microscopy integrated.

Resource	*n*	Category	Method	Outcomes	References
Zoom	20	IV	A, B	Zoom enhanced learner engagement, both students and educators appreciated using Zoom's annotation tool, webcams, and sharing of digitized slides with PathPresenter.^62^ Breakout rooms were technically effective and had pedagogical value, but their impact depended on group size, task design, and session length.^87^ Breakout rooms used for case‐based learning in group was helpful, and visible was the participation in Zoom chat.^54^	[47, 51, 54, 62, 82, 85, 86, 87, 89, 91, 92, 94, 95, 96, 102, 103, 105, 106, 113, 121]
PowerPoint	18	V	A	PowerPoint was used to create the online modules, that overall were evaluated enjoyable, motivating, and valued for their flexibility.^119^ Was used for digitized slide session, 43.1% of the students found PowerPoint presentations helpful.^81^ Used for formative image‐based quizzes in‐class to enhance discussion; students surveys show highly appreciation.^46^ Having the materials (like PowerPoint) of lectures helped students stay on track with the learning^90^	[39, 42, 46, 74, 79, 81, 82, 90, 93, 98, 99, 101, 102, 104, 114, 117, 119, 128]
Moodle	11	VI	A, E	The online module was well evaluated by students who appreciated the friendliness and intuitive interface.^42^ About 74% of the students reported easy to access the environment in Moodle, important factor for an adequate employment of BL^44^	[28, 44, 46, 49, 52, 53, 66, 70, 84, 91, 121]
Blackboard	8	III	A	Blackboard was used to organize course materials and share the syllabus, notes, digitized slides, and lab manual.^83^ About 70% of the students agreed that Blackboard made learning activities easier.^80^ Blackboard was also used to follow up on the attendance^102^	[78, 80, 82, 85, 102, 104, 108, 120]
Microsoft Teams	6	IV, VII	A, D	Microsoft Teams was used to store WSI,^62^ to organize educational materials (lectures, case presentations, and test results),^62^, ^78^, ^104^ and to post and notify students instead of using e‐mails^62^	[62, 78, 84, 101, 103, 104]
MOOCs	6	VI	A, E	The tool effectively combined online and face‐to‐face learning activities, fostering high student engagement and satisfaction.^65^ The MOOC was found helpful their studies from students^112^	[59, 65, 69, 86, 98, 112]
YouTube	5	VIII	E	YouTube was used to store all synchronous e‐learning lessons recorded,^111^ and short videos emphasizing key concept of the lectures.^99^ Students showed a positive perception of the quality of supplementary resources such as videos^100^	[99, 100, 103, 106, 111]
Google Meet	3	VII	A	Google Meet made classes more interactive with screen sharing, audio, video, and chat features, making them more engaging. It also supported multidisciplinary learning by allowing multiple teachers or guest speakers in one session^99^	[68, 99, 103]
WhatsApp	3	VIII	A	WhatsApp was used to submit assignments, and share link to students,^85^ and study materials.^121^ However, students felt that Google Meet was not as suitable for online teaching compared with other tools^121^	[85, 103, 121]
Articulate	2	VI	A	Articulate/Storyline 2 was used to create an online interactive module, and students highly rated it as educational resource^48^, ^76^	[48, 76]
Canvas	2	III	A, C	Canvas was used to store course materials and to submit assignments.^62^, ^96^ It was also used to collect student's surveys^62^	[62, 96]
Google Classroom	2	IV	A	Google Classroom was used for digital lectures^99^, ^103^ and to collect didactic lectures.^99^ It was also used by students to ask questions related to their case‐based learning activities, like ask for specific features or special laboratory tests^99^	[99, 103]
Google Forms	2	VII, IX	A	Google Forms was used to create quizzes with time limits (TIMIFY add‐on),^99^ and with immediate feedback for independent learning.^100^ It also helped to track student progress between tests and to quickly evaluate individual and class performance^99^	[99, 100]
Kahoot!	2	IX	A, D	Kahoot! was used for pools,^82^, ^95^ and used at the end of lab sessions boosted student engagement, motivation, and enjoyment, providing immediate feedback and clarifying misunderstandings to aid examinations preparation^95^	[82, 95]
Online Quiz Primer	2	IX	A, B	The tool was perceived by students as easy to use, user‐friendly, engaging and helpful for training with quizzes^88^, ^113^	[88, 113]
NAVID	2	III	A	NAVID was used for asynchronous learning,^60^ and was considered as an appropriate tool for online learning by both students and educators^49^	[49, 60]
X/Twitter	2	VIII	E	X, formerly Twitter, was used to reach users and promote PathElective.^66^ X, formerly Twitter, was used to create a community and enhance discussion on histology and pathology.^66^, ^97^ Analysis of hashtags and tweets using Symplur showed strong engagement and high impressions, boosting global visibility and real‐time knowledge sharing among professionals^97^	[66, 97]
Amazon Web Services	1	II	A	Amazon Web Service served as a cloud‐based storage solution for digitized slides^106^	[106]
Cisco WebEx	1	IV	E	Cisco WebEx was used to host lectures and virtual conferences during the COVID‐19 pandemic. The use was complemented with links to WSI. The tools was also used to evaluate visitors attendance^97^	[97]
Concentriq	1	II	A	Concentriq, a digital microscopy slide repository by Proscia, was used to access the university website slides collection^106^	[106]
Echo360	1	IX	A, D, E	Echo360 was used to run interactive large‐group sessions at the end of each topic. Students responded to in‐class questions, helping them integrate and reinforce the content covered. It was also used to record lectures^119^	[119]
Facebook	1	VIII	A	Facebook was used with the iSlide platform to support interactive discussions. Students can comment on cases and share annotations, and instructors can provide comments about complicated cases and participate in discussions^57^	[57]
Flexi quiz	1	IV	A	Flexi quiz was used for assessment during the pandemic times of written examinations^85^	[85]
Google Docs	1	VII	A	The tool was used for have collaborative online documents^116^	[116]
Google Drive	1	VII	A	The tool was used to have a shared folder for students to work in group^116^	[116]
Mentimeter	1	IX	A	Mentimeter was one of the app more employed for online formative assessment in Chinese universities during the COVID‐19 pandemic^86^	[86]
OlyVIA	1	I	D	Olyvia was used as digital microscopy software viewer, allowing for panning (navigating) and zooming (magnifying)^120^	[120]
Poll Everywhere	1	IX	A	Poll Everywhere was used for interactive poll sessions. The results indicate strong students support for the tool in relation to their engagement, enjoyment, and morphology understanding^101^	[101]
Rain Classroom	1	III	A	Rain Classroom was used by Chinese universities as teaching management tool (to organize the educational content including videos, presentations, exercises) and to live broadcasting^86^	[86]
Socrative	1	XI	A, D	Socrative was used for online formative assessment. Students appreciated the feedback on their academic performance, though performance differences between online and paper assessments were not statistically significant^129^	[129]
Skyroom	1	IV	C	Skyroom was used for synchronous online learning^60^	[60]
TurningPoint	1	IX	A	TurningPoint was used to engage students with image‐based quizzes. Was particularly useful for the voting handsets feature, which has the advantage of promoting class discussion^46^	[46]
Wimba classroom	1	IV	A	Wimba Classroom was used as a virtual classroom tool to deliver live and recorded lectures, providing also interaction functionalities as “raise hand,” chat area and polling questions^83^	[83]
Xuexitong	1	III	A	Xuexitong was used during the COVID‐19 pandemic by Chinese universities as a teaching management tool and for live broadcasting^86^	[86]

*Note*: Type category: (I) Image viewer software, (II) Digital microscopy cloud services, (III) Learning management system, (IV) Video conferencing tool, (V) Presentation software, (VI) Online interactive modules, (VII) Collaborative platforms for group working, (VIII) Discussion board, (IX) Interactive live quizzes, (X) E‐books, (XI) Others. Methods: (A) Survey/Online questionnaire ‐satisfaction, acceptance‐, (B) Usability testing, (C) Student learning metrics ‐grades, completion rates‐, (D) Student diagnosis performance, (E) User behavior‐login info, views, traffic data, interaction rates.

**TABLE 5:** Digital learning tools with digital microscopy integrated.

Resource	Organization	URL	Accessibility	Subject	Functionalities found in reviewed articles
BEST slice^78^	BEST Network	https://slice.best.edu.au/	Access with credentials	Histology and pathology	Cloud‐based collection of WSI, Annotations from both educators and students, Image‐based quizzes with instant feedback, Supplementary educational content
cLovid^54^	University of Turku, Finland, UMC Utrecht, the Netherlands, University of Münster, Germany	https://clovid.org/	Open access and Open source	Pathology	Collection of WSI, Annotations, Image‐based quizzes with instant feedback, Clinical cases, Collaborative learning features, Supplementary educational content
Cytomine^45^, ^65^	University of Liège, Belgium	https://cytomine.com/	Open access and Open source	Histology	Collection of WSI, Annotations, Collaborative learning features, Learning analytics
Digital SlideBox^46^	University of Bristol, United Kingdom	https://slidebox.bristol.ac.uk/	Access with credentials	Histology	Collection of WSI, Annotations, Student personalized annotations, Image‐based quizzes with instant feedback, Supplementary educational content
HistoNFC^109^	University of Cordoba, Spain	Through Near Field Communication (NFC) technology	Mobile access utilizing NFC tags associated with glass slides	Histology	Collection of static images, Supplementary educational content
HistoViewer^71^	Aarhus University, Denmark	https://histoviewer.biomed.au.dk/	Open access (registration required)	Histology	Collection of WSI, Annotations, Comparison module with multiple slides, Collaborative learning features
Histologie für Mediziner^43^	Goethe University Frankfurt, Germany	https://tinygu.de/histodemo (Demo)	Access with credentials	Histology	Modules with annotated static images, Quizzes with instant feedback, Supplementary educational content
Histology Laboratory Manual^55^	University of Central Florida, United States	Not found	Access with credentials	Histology and pathology	Collection of WSI, Annotated static images, Quizzes with instant feedback, WSI‐based clinical cases, Collaborative learning features
iSlide^57^	Taipei Medical University, Taiwan	http://www.dermpath.org.tw/EN/digital‐slides/islide.html (Demo)	Not defined	Pathology	Collection of interactive WSIs, Supplementary educational content, Clinical cases
Kurt's Notes^73^	Dr. Kurt Schaberg, University of California, United States	http://kurtsnotes.net/	Open access	Pathology	Collections of PDF notes with annotated static images and infographics, Image‐based quizzes, WSI‐based clinical cases, Flashcards, Supplementary educational content
Michigan Histology website^52^, ^74^, ^79^, ^93^, ^100^	University of Michigan Medical School, United States	http://histology.medicine.umich.edu/	Open access	Histology	Collection of WSI, Collection of EM static images, Annotated static images, Image‐based quizzes with feedback, Supplementary educational content
MAPA^53^	George Washington University, United States	http://microanatomyatlas.com/	Open access (registration required)	Histology and pathology	Collection of WSI, Annotations
MyMi.mobile^47^, ^61^, ^89^	University of Ulm, Germany	https://mymi.uni‐ulm.de/	Access with credentials	Histology	Collection of WSI, Annotations, Image‐based quizzes, Learning analytics
Pate^40^	University Medical Center and Johannes Gutenberg University, Germany	http://pate.um‐mainz.de/ (Not working)	Not defined	Histology and pathology	Collection of interactive WSI, Annotations, Supplementary educational content
PathElective^58^, ^66^	Loyola University and more USA University, United States	https://www.pathelective.com/	Open access (registration required)	Pathology	Modules with video and digital resources based on WSI or static images, Certificates and tracking progress of course completion
pathCast^28^	Mount Sinai West Hospital, United States	http://pathologycast.com/; https://youtube.com/c/pathCast	Open access	Pathology	Live streaming and archived lectures based on WSI or static images, Access to expert‐led lectures from various institutions worldwide
PathoDiscovery^41^	Radboud University Medical Center, The Netherlands	https://radboudumc.bkcacademy.nl/module/19/extern (Demo)	Not defined	Histology and pathology	Collection of WSI, Annotations, Image‐based quizzes, Supplementary educational content
Smartpathk^38^	Federal University of Piauí, Brazil	http://smartpathk.pmadt.com.br/	Access with credentials	Pathology	Static images clinical cases, Image‐based quizzes with automatic generation of quizzes using machine learning decision tree model
PathPresenter^62^, ^66^, ^97^	Company made by academic pathologists	https://www.pathpresenter.com/	Open access (registration required)	Pathology	Cloud‐based collection of WSI, Annotations, Image‐based quizzes and case studies, Supplementary educational content, Upload custom WSIs to create and share personalized quizzes and cases
The digital microscope^65^	University of Namur, Belgium	https://www.histology.be/	Open access	Histology and pathology	Collection of WSI, Supplementary educational content
Virtual Microscopy Database (VMD)^25^	Community of histology educators' members of American Association for Anatomy (AAA)	http://www.virtualmicroscopydatabase.org/	Open access (registration required)	Histology and pathology	Collection of WSI, Upload custom WSIs to create and share personalized quizzes and cases
Virtuelle Histokasten Jena^72^	Friedrich‐Schiller‐University Jena, Germany	http://www.anatomie2.uniklinikum‐jena.de/Studium/Virtueller+Histokasten.html	Access with credentials	Histology	Collection of WSI, Collaborative annotations, Modules and tutorials with static images annotated, Supplementary educational content
Virtuelle Mikroskopie^87^	Westfälische‐Wilhelms‐University, Germany	https://mikroskopie‐uds.de/	Open access	Histology	Collection of WSI, Annotations, Supplementary educational content
WebPath^56^	Edward C. Klatt, Mercer University School of Medicine, United States	https://webpath.med.utah.edu/	Open access	Pathology	Collections of annotated static images and infographics, Quizzes with average score, Clinical cases, Supplementary educational content

*Note*: The list of digital learning tools with integrated digital microscopy reflects the information identified by the authors during the review of the included articles. “Subject” indicates the discipline the tool was used for in the included studies.

### Assessment methods

The theme “Assessment Methods” covered various approaches for evaluating student knowledge in blended or digital learning environments. Table 6 lists the different assessment methods identified in the articles, including how often each method appeared. The most frequently reported methods were quizzes with multiple choice questions (MCQs), with some studies also mentioning open‐ended questions. Other frequently reported assessment methods included written examinations, team‐based assessments, and oral examinations.

**TABLE 6:** Categorization of assessment methods found in the included articles.

Assessment method	Definition	*n*	References
Quizzes	Multiple choice questions (MCQs), image‐based MCQs; few studies also included open‐ended or unspecified question types	37	[27, 28, 39, 43, 45, 46, 48, 50, 51, 55, 56, 60, 65, 73, 74, 76, 79, 82, 83, 86, 87, 88, 93, 94, 95, 99, 100, 101, 102, 112, 113, 114, 117, 118, 119, 120, 122, 124, 129]
Written examinations	A traditional written examination format combining MCQs and short essay questions (SEQ) for comprehensive assessment	13	[45, 46, 65, 69, 70, 76, 83, 85, 91, 100, 104, 114, 129]
Team‐based assessments	Group presentations of case studies, projects, and problem‐solving tasks	7	[55, 69, 96, 100, 112, 114, 122]
Oral examinations	A verbal examination to demonstrate knowledge, reasoning, or communication skills	5	[50, 71, 75, 82, 104]
OSPE (Objective, Structured, Practical Examination)	A timed, structured practical examination conducted online, used to evaluate practical skills	4	[85, 91, 102, 129]
Assignments	Homeworks or individual tasks	4	[85, 86, 87, 100]
Practical lab examinations with light microscope	In‐person practical lab examinations using light microscopes	3	[83, 118, 125]
Presence	Student attendance online or in‐person lectures	3	[68, 86, 87]
Peer assessments	Provide feedback to other students	3	[51, 75, 86]
Class participation	Active engagement in class discussions and activities	2	[68, 100]
Reflective journals, portfolios, and/or essays	Written tasks where students document their learning process, experiences, and insights	2	[96, 125]
Hand drawing	Hand drawings of cellular structures	1	[83]

### Interactions between students and educators

The theme “Interactions between students and educators” covered the various forms of communication and relational exchanges that occur within educational settings, including verbal and non‐verbal communication. Table 7 lists the different categorizations of interactions between students and educators identified in studies, including how often each method appeared in the articles. Physical meetings and chatting during online lectures were the most frequent forms of interaction, followed by video conferencing and email communication.

**TABLE 7:** Categorization of interactions between students and educators found in the included articles.

Interactions between students and educators	Definition	*n*	References
Physical meeting	In‐person meetings between students and educators	10	[44, 46, 65, 70, 83, 87, 105, 112, 114]
Chat during online lectures	Real‐time text‐based interactions	9	[59, 62, 79, 80, 83, 87, 90, 100, 112]
Video conferencing meeting	One‐on‐one or small group meeting online	6	[51, 82, 87, 90, 96, 105]
Email communication	Communication on emails	6	[51, 82, 87, 90, 98, 117]
Discussion forum	Asynchronous platforms for posting questions, comments, and discussions	4	[51, 82, 90, 117]
Feedback on assignments	Feedback on assignment with e‐mentoring	1	[100]

### Traditional versus digital and blended learning

The theme “Traditional versus digital and blended learning” focused on the perceptions of students and educators about the transition from traditional to digital or blended learning. Table 8 presents the perspectives of students and educators, along with the data collection methods used. Many studies reported that students and educators appreciated the flexibility and accessibility associated with digital approaches. Reduced engagement and technical limitations were among the challenges reported in relation to digital learning.

**TABLE 8:** Perceptions of students and educators on the transition from traditional to digital or blended learning.

First author, year	Method of data collection	Subject	Perception
Abdelbagi 2023^80^	A	Pathology	The article reported students' positive perception of online pathology teaching during the COVID‐19 pandemic. Recorded pre‐lecture videos created by teachers significantly aided students' understanding. They appreciated the support provided by teachers throughout the pandemic
Agarwal et al. 2019^37^	A	Pathology	The article found that most students preferred digitized slides on Blackboard over light microscopy for learning pathology lesions, appreciating the flexibility it offered. However, some missed the direct teacher interaction
Almohammadi 2024^81^	A, E	Pathology	The most valued feature of digitized slides was their accessibility across different times, locations, and devices. While 51.7% of students enjoyed the digital lab experience, 83.5% expressed a preference for a combination of glass slides and digitized slide classes
Aristotle et al. 2021^117^	A, F	Histology	The blended flipped classroom method showed statistically significantly higher post‐test scores compared with traditional teaching (*p* < 0.0001). Student feedback was very positive. Blended FC enhanced understanding, problem‐based learning, and competency achievement
Avilova et al. 2022^82^	A	Histology	In the two universities, the experience of online education is positive overall, with challenges in internet connection and a lack of laboratory‐based teaching. Students agreed that the main advantages were the extra time they had to prepare for classes and prerecorded lectures
Barbeau et al. 2013^83^	E	Histology	There were not significant differences between online laboratory course and a traditional one in grades and performance. The online format has expanded the enrollment beyond the physical lab's capacity and allowed a summer version with minimal impact on faculty
Başer et al. 2024^84^	A, F	Histology	This study aimed to investigate the impact of digital microscopy and LM on the satisfaction of medical students and how they affect student performance. LM scored higher in the questionnaire (*p* = 0.010), but digital microscopy showed higher examinations averages (*p* = 0.013)
Bhardwaj et al. 2021^85^	A	Histology	The results found that students had a good acceptance toward online learning, appreciating the flexibility and enhanced engagement it offers. However, students faced limited hands‐on practice, poor technical support, and less interaction with peers
Cheng et al. 2021^86^	A	Histology	A survey of various Chinese universities during the pandemic found that first‐tier and new first‐tier cities adapted more effectively to online learning due to stronger digital resources and IT support. Enhancing teacher–student interaction was a challenge, while digital microscopy proved highly effective for practical sessions, with around 35% of schools maintaining its use unchanged
Cheng et al. 2017^118^	A, F	Histology	The results indicated strong student support for the flipped classroom, with positive engagement and interest. Participants showed improved learning outcomes. Teachers had more time for discussions and quizzes instead of just lecturing, which helped students in the flipped classroom do better. This study suggests flipped learning can improve histology education
Cosnita et al. 2020^124^	D, E, F	Histology	The article highlighted the benefits of building an e‐assessment system. Students liked instant feedback in both the assessment module and after the examinations, decreasing complaints about methodology and stress while waiting for results
Darici et al. 2021^87^	A, E, G	Histology	The digital histology course was well‐received, with students favoring multiple choice questions and digital microscopy for examinations preparation. However, senior students performed worse. Moreover, some students became passive participants, partly due to limited social interaction
Ettarh 2016^114^	A, F	Histology	Students had lower satisfaction with the hybrid model compared with traditional labs, preferring structured, faculty‐led sessions. However, they were more engaged, with higher attendance and improved performance on the examinations during hybrid format, indicating effective learning despite mixed reception. From the instructors' viewpoint, the hybrid format encouraged teamwork, critical thinking, and alignment with medical training goals
Fermozelli et al. 2017^44^	A	Pathology	The results found that the majority of students responded positively to blended learning, reporting increased motivation and improved contextualization of pathological concepts. It allowed students to connect pathology more effectively with practical medical applications
Gatumu et al. 2014^46^	A	Histology	Students preferred digital microscopy for its accessibility, ease of use, and annotation features, which allowed learning beyond the lab through quizzes and assessments. However, some expressed concerns about losing traditional LM skills
Gellisch et al. 2022^89^	A, H	Histology	The study found that face‐to‐face learning was associated with stronger stress responses compared with online learning. This was evidenced by a significant reduction in heart rate variability and higher cortisol levels in face‐to‐face settings. Practical microscopic anatomy in online environment suggested lower stress levels for medical students
Gellisch et al. 2023^47^	A, H	Histology	The study compared face‐to‐face learning, regular online learning, and interactive online learning. Results found that the physiological arousal of students engaged in online learning can be enhanced via interactive teaching methods, and higher engagement was linked to better attention and learning
Gonzalez Donoso et al. 2023^90^	A	Histology	Results were used to define three factors to measure student participation in e‐learning histology course: 1. Habits of online, 2. Motivation for online learning, 3. Interaction of online. Students generally perceived their engagement in the e‐learning histology course positively. They reported high motivation, especially through interactive components like discussions and chats with instructors
Hanafy et al. 2021^91^	A, E	Histology	Traditional teaching was perceived as more effective by students and staff that preferred it for support in collaboration, critical thinking, and structured guidance. However, online examinations offered the benefit of instant feedback but also posed a higher risk of cheating
Johnson et al. 2015^93^	A	Histology	Medical students favored digital resources over traditional textbooks, with extensive usage of online course site and practice questions. They appreciated independent study and autonomy in learning
Kafle et al. 2022^94^	A	Pathology	Students recommended including the e‐learning clinical pathology in the curriculum, showing strong support for this vertical integration approach to enhance diagnostic skills and patient management
Kashif et al. 2023^130^	A, F	Pathology	Results indicated that blended learning, which combined digital resources with interactive discussions, led to higher student engagement and performance. The study highlighted the advantages of blended learning for fostering a deeper, more interactive learning experience and suggests its effectiveness over traditional or solely digital methods in medical education
Khan et al. 2023^131^	A, F	Pathology	The study compared computer‐assisted learning with conventional chalk‐and‐board methods, both conducted in‐class. Results showed that the computer‐assisted group achieved higher scores and that students found it easier to understand and remember diagnostic features
Lieberman et al. 2021^96^	A, D	Pathology	The course shifted from a 2–4 student in‐person rotation to a 40‐student fully online format during the COVID‐19 pandemic, emphasizing interactivity and small group work. This change improved visibility and access to lab medicine education, including for out‐of‐state students. Despite some teleconferencing fatigue, students reported higher engagement and overall approval for the online format
Mantaring et al. 2023^108^	A	Histology	The study found that medical students using LM showed a more positive attitude and higher satisfaction in using this traditional method. No performance difference between digital microscopy and LM group were found
Mastour et al. 2023^60^	F	Pathology	The students performed better in digital format than in the in‐person (*p* < 0.05). The study concluded that e‐learning can be a successful implementation and can be used as an alternative medical educational method
Medina et al. 2019^109^	A	Histology	The HistoNFC system provided on‐demand access to information and supported didactic activities. Medical histology students highly valued the use of this technology and its application in the course
Mukhopadhyay et al. 2021^99^	A	Pathology	The article highlighted a successful transition to digital microscopy teaching during the COVID‐19 pandemic, showing improved student engagement and performance with online case‐based learning. Despite connectivity challenges for some, the study supports integrating online tools with traditional methods for effective medical education
Naseer et al. 2022^129^	A	Histology	Students performed slightly better in online formative assessments than in traditional examinations, though the difference was not statistically significant (*p* = 0.307). Most students were satisfied with online learning resources and felt supported by their teachers. A combined blended learning approach is recommended for the future
Nikas et al. 2022^100^	A	Histology and pathology	Out of 255 students, 68% responded, showing positive views on online learning. Students actively participated using interactive tools and digital microscopy for lab practice. Findings suggested that most students would prefer a blended learning approach for the future
Otifi et al. 2023^102^	A, E	Pathology	About 60% of students found e‐learning valuable, and 84% want it to continue after the pandemic. Test scores improved after digital learning. However, some students stated that their academic progress was negatively influenced by e‐learning
Paul et al. 2022^103^	A	Histology	The study revealed key challenges in online anatomy and histology education during the COVID‐19 pandemic, with 47% of educators facing unstable platforms, 26% struggling with unfamiliarity with online tools, and 17% lacking adequate training and support
Pesesse et al. 2023^65^	A, E, F	Histology	Student appreciated the integration of an e‐learning module with classroom‐based teaching. Students overall were satisfied with the quality of the e‐module and the face‐to‐face activities. The level of performance was maintained in the educational strategy implemented
Prados‐Carmona et al. 2022^122^	A	Pathology	Results found that 77% of the students rated 8/10 the mobile app. The findings suggested that the app can serve as an effective support tool, facilitating the acquisition of clinical skills in a more flexible and accessible way
Rodrigues et al. 2022^68^	A	Pathology	The results reported students' positive reception of remote teaching, valued as positive by 80% of the students. Live interactive sessions were particularly valued, even suggested as replacements for traditional in‐person classes
Ruan et al. 2022^69^	A, F	Histology	Test scores among students who had taken the blended prerequisite course online, were better than in the non‐prerequisite course group. The blended learning approach made the experience more engaging and effective
Schmidt 2013^72^	A	Histology and pathology	Survey results showed only 5% of students regularly used LM for independent study, while digital microscopy had higher engagement: 67 out of 172 students frequently used “HistoInteraktiv,” and 65 used it very often
Singh et al. 2023^104^	E, F	Pathology	The marks obtained by the students who attended online pathology classes during the COVID‐19 pandemic in 2020–2021 session were compared with previous group 2019–2020. The online group scored higher marks than traditional group in theory and practical (*p*‐value < 0.05)
Somera Dos Santos et al. 2021^125^	A, E, G	Histology	This study found that students prefer digital microscopy over traditional LM due to its practicality and effectiveness. However, some students expressed concerns about LM skills, crucial for medical practice, which can be addressed by maintaining occasional LM access. No significant difference was observed in scores
Tanaka et al. 2021^105^	A	Histology and pathology	The remote pathology elective received a high rating (4.88 out of 5), reflecting students' positive experiences. Feedback indicated the elective enhanced flexibility and diagnostic skills. However, few students noted challenges with focus in the digital format and missed social connections
Tauber et al. 2019^75^	A	Histology	The results of the questionnaire indicated that 100% of the students preferred digital microscopy compared with traditional LM. They also frequently used digital microscopy system for preparation of examinations
Tecson et al. 2023^110^	A, F	Histology	This study looked at a blended program called LEAP that helped medical students improve their anatomy and histology skills after online learning during the COVID‐19 pandemic. Students had higher acceptance of the program, especially for using traditional methods and human anatomical specimens over digital tools. Test scores improved after the program, and students also showed positive behavior and attitudes
Tina et al. 2024^126^	A, F	Histology and pathology	The group taught with the digital microscopy scored significantly higher on tests compared with the group taught with LM. The majority of students responded positively to the digital microscopy usage, citing improvements in understanding and engagement. Many students agreed or strongly agreed that digitized slides were helpful in revising normal anatomy and histology and improving clinico‐pathological correlations
Waugh et al. 2022^78^	A, F	Histology and pathology	Second‐year medical students found online histopathology more comfortable and better structured than in‐person teaching. No changes in individual performance compared with the previous year of only in‐person teaching (*p* = 0.30)
Wu et al. 2023^107^	A, F	Histology	The study found strong student acceptance for blended lectures (81.13% of students). Online classes were rated as user‐friendly (83.02%) and effective for learning (80.19%)
Zalat et al. 2021^132^	A	Histology and pathology	Most staff (88%) agreed that online course skills enhanced educational experience. Agreement on e‐learning usefulness, ease of use, and acceptance was 77.1%, 76.5%, and 80.9%. Key barriers included poor internet (40%), inadequate labs (36%), and lack of devices (32%)
Zhong et al. 2022^112^	A, F	Histology	Blended flipped classroom in physical classroom students scored higher on final examinations and were more likely to engage in face‐to‐face interactions with instructors. Students participating in physical or digital blended learning scored better in quiz than the students from the traditional learning group
Zureick et al. 2018^79^	A, F	Histology	Students attending lectures, live or via video, scored better in histology than those mixing both. Distractions like social media or interruptions lowered scores. While video lectures grew popular for flexibility, staying focused was key. Faster playback did not impact learning

*Note*: Type: (A) Survey/Online questionnaire ‐satisfaction, acceptance‐, (B) Interviews, (C) Focus group, (D) Observation, (E) Students learning metrics, (F) Student diagnosis performance, (G) User behavior ‐login info, views, traffic data, interaction rates., (H) Physiological stress parameters ‐heart rate variability, salivary cortisol concentration‐, and (I) Other. “Subject” indicates the discipline the articles mentioned in the included studies found by the authors.

## DISCUSSION

This review explored various teaching methods, digital learning tools, assessment methods, student and educator interactions, and perspectives on the transition from traditional to digital and blended learning in histology and pathology education for medical students. The inclusion of various study designs and the selected timeframe, spanning from January 2013 to September 2024, captured the transition from traditional microscopy to the use of digital microscopy and digital tools in teaching. Although this process began earlier in some institutions, we focused on the past decade to highlight recent progress. In the discussion, recent literature that was not identified in the systematic literature search was added to critically analyze the findings.

Over the last 10 years, there has been a steady increase in the number of publications regarding the use of digital microscopy in histology and pathology teaching, particularly following the COVID‐19 pandemic. This indicates a growing interest in the topic in recent years and suggests continued interest in the future. The data showed that the publications focused more on histology than pathology, and thus primarily on medical students in their early years of education, as learning the characteristics of normal tissue (histology) is the foundation for understanding pathology.

The following sections synthesize the findings from all included studies, highlighting trends, benefits, and challenges associated with the integration of digital microscopy and digital teaching methods into histology and pathology education.

### Teaching methods

Among the most frequently cited teaching methods were those based on digital interaction, e‐learning modules, and virtual classrooms. Technological advances, curriculum changes, and reduced teaching time have contributed to a shift in pedagogy in health care education.^5^, ^133^ Many institutions have increasingly explored hybrid teaching methods and self‐directed learning,^134^, ^135^ as is reflected in the findings of this study on histology and pathology education. The integration of digital microscopy and online tools has opened up new possibilities for more accessible, interactive, student‐centered and active learning experiences.^136^, ^137^, ^138^, ^139^

Several studies^39^, ^51^, ^80^, ^82^, ^117^ underlined the benefits of pre‐lesson materials, such as digitized slides, video lectures, and interactive modules, allowing students to learn at their own pace. Recorded lectures were identified as effective in supporting autonomous learning, with students considering the ability to revisit content one of the main advantages of online learning.^82^ However, this flexibility comes with a trade‐off. Some studies found that the availability of recorded lectures has reduced student attendance at in‐person lectures,^140^, ^141^ which was associated with lower performance and final grades.^142^, ^143^ However, more recent studies have shown that there is no significant correlation between attendance and academic performance.^144^, ^145^, ^146^ Zureick et al.,^79^ found that students' performance was not tied to whether students attended class or used digital methods, but rather to students' consistency and ability to avoid distractions.

Flipped classroom, where students are encouraged to self‐study before class and engage in activities during class, has been shown to improve student performance, engagement and satisfaction in histology education.^65^, ^112^, ^117^, ^118^ Other studies also show that flipped classrooms can foster engagement and performance in early medical training.^147^, ^148^ Combining flipped classroom, problem‐based learning, case‐based discussion, and collaborative exercises was found effective for pathology teaching.^44^, ^62^, ^70^ The ability to understand clinical case analysis and contextualize histopathological processes is important for the development of medical students' critical thinking. A combination of active learning activities appears to better help educators support the development of these competencies.^44^, ^149^ Several studies show that integrating digital microscopy and online resources into active teaching methods can play a key role in enhancing students' motivation to learn and understand microscopic structures in histology and pathology courses.^112^, ^150^, ^151^

Team‐based learning (TBL) supported by digital microscopy tools was reported in several studies, in histology^65^, ^71^, ^114^ and pathology^53^, ^54^, ^55^ teaching. TBL activities foster collaboration and clinical relevance in pathology education,^152^, ^153^ reflecting how medical curriculum has become more integrated.^133^, ^154^ Parker et al.^62^ used “Detective Case” and “Good Will Hunting Case” in an online pathology course. In this course, students engaged in team‐based activities with digitized slides. In “Detective Case,” students worked collaboratively to solve a case, presenting their diagnosis and reasoning. In “Good Will Hunting Case,” students were asked to interact with expert consultants to request tests and solve a complex diagnostic case. Cross‐disciplinary TBL, involving pathologists and clinicians, can provide students with holistic understanding of diagnostic processes particularly in cancer diagnostics and management.^105^

Game‐based learning also emerged as a promising method in histology^87^, ^95^, ^114^ and pathology^62^, ^68^ teaching. Ramya et al.^115^ used crossword puzzles and mind maps to reinforce students' understanding of complex concepts. “Histopoly,” a digital board game designed to study tissue types using quizzes, drawings and presentations, increased students' satisfaction and peers interaction.^155^ “They Know,” a digital game used to test anatomy and histology knowledge, helped students identify knowledge gaps and strengths.^156^ Art has also been used to gamify histology teaching. In Cimpean et al.,^157^ students were asked to participate in a competition and use digitized slides to create artwork. By creating a more relaxed and supportive learning environment,^158^ game‐based activities were found to increase motivation and participation in histology and pathology education.^159^, ^160^

The findings of this section highlight a pedagogical shift in histology and pathology teaching. Active learning, personal autonomy, collaboration, and self‐determination emerged as key components, supported by methods such as flipped classroom, TBL, problem‐based, case‐based, and game‐based learning.

### Digital learning tools

The introduction of digital microscopy in histology and pathology education opened new possibilities in the development of digital learning tools. Educators in several institutions have adapted existing software and learning management systems (LMS) to store and present digitized slides.^37^, ^64^, ^81^, ^84^, ^128^ Access to digitized slides and their use in histology and pathology education can make medical students be more comfortable in using digital microscopy‐based tools in their future careers and clinical workflows.^161^ Moreover, students themselves may play a vital role in shaping digital learning tools. In Hayburn et al.^48^ students created an interactive tutorial with digitized slides for their peers for anatomy and histology teaching. The study highlighted the importance of involving students in co‐creating educational materials, as it increases engagement for their learning process.

Web‐based learning tools with digital microscopy or static images integrated, and repositories of digitized slides, now play a central role in histology and pathology education. Digital microscopy‐based tools were developed and used well before the pandemic,^43^, ^46^, ^53^, ^55^, ^57^, ^67^, ^72^, ^74^, ^79^ but recently the use of such tools has grown significantly.^39^, ^41^, ^43^, ^45^, ^47^, ^52^, ^54^, ^58^, ^59^, ^61^, ^65^, ^66^, ^73^, ^78^, ^87^, ^89^, ^97^ Some digital tools were not covered in scientific articles, and they were therefore not identified in the systematic literature search presented in this study.^162^, ^163^, ^164^, ^165^, ^166^, ^167^, ^168^, ^169^, ^170^, ^171^, ^172^, ^173^, ^174^, ^175^

Some digital learning tools provided static images of histopathological sections with a description of the images,^43^, ^56^, ^170^ while others integrated WSI viewers that allow users to explore digitized slides interactively by zooming and navigating.^46^, ^52^, ^53^, ^54^, ^71^, ^87^, ^162^, ^164^, ^165^, ^166^, ^171^ An advantage of having a digital learning tool with a WSI viewer is the ability for educators to add annotations on the WSIs. Such instant digital information may help students learn how to recognize features and structures in the tissue sections,^176^ understand unclear topics, and make teaching more efficient for students and educators, thus reducing teaching time for the educators.^151^, ^177^ This can aid students in practicing their microscopy skills independently, thus supporting self‐directed learning.^22^, ^178^

Different needs for novice or senior students should be considered in the design of digital learning tools.^150^ Meyer^22^ reported that some students found annotations distracting. A function that can turn annotations on and off can let students identify given structures by themselves and be helped when needed.^22^, ^176^ More experienced learners benefited from more complex quizzes and diagnostic scenarios.^179^ A comparison of different WSIs or multi‐magnification comparisons was found to help students strengthen their understanding of tissue morphology.^71^

Using digital learning tools with digital microscopy integrated requires costly infrastructure, software, and maintenance. Some institutions have developed open‐access systems, allowing students and educators worldwide to use them.^25^, ^28^, ^45^, ^52^, ^54^, ^56^, ^65^, ^66^, ^71^, ^73^, ^162^, ^163^, ^164^, ^165^, ^166^, ^167^, ^168^, ^169^, ^170^, ^171^, ^172^, ^173^, ^174^, ^175^ Open‐access solutions can provide high‐quality educational material to less developed countries or institutions with scarce resources, overcoming cost barriers associated with digital microscopy.

Social media has been used in histology and pathology education for students' self‐studies,^72^ and to share digitized images or videos that demonstrate WSI assessment.^28^, ^97^ Several institutions and educators have opened social media accounts to share histological images, annotations, diagrams and quizzes with students.^180^, ^181^, ^182^ Video‐sharing platforms, such as YouTube, can help students learn from experts that guide them in scanning and interpreting digitized slides, using visual and audio narration.^1^, ^10^, ^183^ Social media platforms can be used by experts and trainees to exchange ideas and content, fostering collaboration, interaction, and discussion.^184^ As a supplement to student–educator interactions, integrating social media into microscopy classes was found to help students become more active learners.^183^

Mobile applications were also used as digital learning tools with digital microscopy integrated for histology and pathology teaching.^109^, ^122^, ^185^ Several institutions built mobile applications such as SecondLook™^186^ and MyMi.Mobile,^187^ providing students access to collections of digitized slides, enabling self‐studying and self‐testing using annotations and quizzes. HistoNFC^109^ used Near Field Communication (NFC) chips embedded in glass slides. NFC chips allow devices to exchange data when they are in close proximity, making students able to access digitized slides, quizzes, and learning material through a web app. As no installation was required, it offered quick and user‐friendly access to histology content. Using mobile applications allowed students to access and review digitized slides and annotations anytime and anywhere.^188^, ^189^ However, it can also lead to distractions and superficial learning.^190^ The multitude of applications available can make it difficult for students to identify trustworthy learning tools.^189^, ^190^ Institutions and educators should guide students in choosing and integrating digital tools in their education.^189^, ^191^

Flashcards have been used by many students for self‐studies and testing.^192^ Flashcards used for learning histology and pathology can be physical, such as Netter's Histology Flash Cards,^193^ or digital, such as the decks made available in Kurt's Notes.^194^ Students can also auto generate their own deck of flashcards, using apps like Anki or Quizlet.^195^, ^196^ The use of such resources reflects students' active role in learning, and the importance of engaging them in activities that involve testing, repetition and recalling,^197^ improving retention of histology and pathology.

The findings of this section highlight the increasing role of digital learning tools in histology and pathology education. Digital microscopy, together with mobile applications, flashcards, and social media platforms, support flexible, self‐paced, and interactive learning experiences. Opportunities and challenges related to accessibility, engagement, and pedagogical alignment were underlined. These findings reflect broader literature in higher education,^198^, ^199^ where technology continues to transform how students learn and interact with educational content.

### Assessment methods

The use of digital microscopy in histology and pathology education has had an impact on medical student assessment methods. Quiz questions integrating digital tissue slides were mentioned in several studies.^45^, ^46^, ^55^, ^56^, ^65^, ^71^, ^74^, ^76^, ^79^, ^93^, ^100^, ^101^, ^102^, ^104^, ^112^, ^128^, ^129^ Their use enabled students to engage with visual content, potentially improving their diagnostic and tissue recognition skills.^22^ Formative assessments, such as continuous feedback, self‐evaluation, and reflective learning were widely mentioned in the literature.^45^, ^46^, ^55^, ^56^, ^60^, ^62^, ^65^, ^71^, ^74^, ^76^, ^78^, ^88^, ^95^, ^100^, ^101^, ^118^, ^119^, ^128^, ^129^ These approaches aim to promote self‐regulation and agency, which means that students can take ownership of their academic growth and learning.^200^, ^201^

Students reported enjoying the use of formative quizzes to test their knowledge, practice for examinations, and receive educators' feedback.^65^ With tools like Kahoot!^82^, ^95^, ^202^ or other live quizzes that provide instant feedback on quiz results,^48^, ^99^ students can assess their ability to recognize cellular structures in digitized slides and thereby increase a sense of mastery. Instant digital feedback is also of great value to the educators, as it makes teaching more efficient by reducing the number of individual student–educator interactions.^203^, ^204^ Thus, digital feedback may help educators handle a higher number of students simultaneously, potentially reducing the time they spend on teaching.

Summative assessments, however, remain crucial for final evaluation and certification. Several institutions integrated digital tissue slides into their questions in summative examinations.^46^, ^124^, ^177^ In Cosnita et al.,^124^ an examination module with WSI and automatic correction of examinations was developed. Initially, educators expressed concerns related to the transition to an automatic system, but were satisfied with spending less time on examinations. The students reported reduced stress levels and appreciated getting immediate feedback on their examination results. Other studies reported that immediate feedback was beneficial for retention of knowledge.^205^, ^206^ To overcome cheating in the online environment, Mastour et al.^60^ used live online videos during examinations to identify suspicious behavior, such as repeatedly glancing away from the screen. A back‐up solution may be needed in case of technical challenges during the examination.^46^

This section highlights the growing integration of digital microscopy into assessment practices in histology and pathology education. Assessment methods such as quizzes and formative feedback help students engage with visual material, improve recognition skills, and support self‐directed learning. Summative assessments integrate digitized slides and automated grading systems, improving efficiency and consistency. These findings align with the broader shift in higher education toward more interactive and learner‐centered assessment methods that benefit both students and educators.^207^, ^208^

### Interactions between students and educators

Digital learning offers flexibility, convenience and autonomy in learning,^44^, ^46^, ^85^, ^90^, ^93^ however, a downside of using fully digital teaching methods is the lack of student–educator interactions.^37^, ^80^, ^85^, ^86^, ^87^, ^91^, ^105^, ^209^, ^210^ Although technology offers unprecedented opportunities for remote interaction and self‐directed learning, students reported missing physical and direct interactions with educators and peers.^37^, ^105^ To ensure interactions between peers, a blended environment has been found to offer good balance.^44^, ^114^, ^130^ A combination of digital resources and in‐class interactive discussions and activities was reported as effective for improving learning outcomes and understanding by educators and students.^107^, ^112^ Physical meetings may be necessary to develop the students' sense of professional identity, and help them develop communication skills that are needed to understand and describe the morphology in histopathological sections.^105^, ^211^

Some software and digital learning tools have features that mimic the traditional flow of a lecture, to make digital classes feel like physical lectures. In Barbeau et al.,^83^ educators appreciated a software interface that enabled monitoring student attendance. Polls can be added, and the students can raise their hands. When students responded to polls, results can be displayed immediately. Moreover, in Darici et al.,^87^ educators reported that the “breakout rooms” function facilitated team‐based work and interaction with peers and educators.

The integration of digital microscopy and digital learning in education of histology and pathology is relatively new due to the recent technological advances of digitization of glass slides and infrastructures that can handle the large files.^212^ Training educators in teaching in digital and blended environments may improve learning experiences for students. According to Adbelbagi,^80^ the extensive training provided to educators increased student satisfaction and their sense of support during online teaching.^80^ In some studies, students reported good interaction in the digital setting through discussion and chats with instructors.^68^, ^90^, ^100^ If social interaction is not integrated into the online course, some students may become passive rather than active participants.^87^

This section highlights that while digital learning increases flexibility and autonomy, it can reduce direct interaction between students and educators. Incorporating interactive features, such as polls, breakout rooms, and discussion tools, along with training educators in digital pedagogy, can help improve student satisfaction and engagement. Institutions should also provide best practices to improve the quality of student–educator interactions.^213^, ^214^ These findings emphasize the importance of maintaining meaningful interactions and combining digital tools with in‐person activities. Such approaches are essential for supporting student learning in digital or blended learning environments.

### Traditional versus digital and blended learning

The comparison of traditional versus digital and blended learning methods in histology and pathology education gave insight into student engagement, learning outcomes, and preferences. Several studies found that digital learning led to better results and scores in quizzes and final examinations for medical students, for both histology^65^, ^131^ and pathology teaching.^60^, ^104^, ^131^ Gellisch et al.^47^, ^89^ found that face‐to‐face learning environments were associated with stronger stress and physiological arousal responses compared with online learning. In acceptance surveys, students generally gave positive feedback regarding online learning.^68^, ^80^, ^82^, ^85^, ^87^, ^90^, ^93^, ^94^, ^96^, ^99^, ^100^, ^105^, ^107^, ^109^, ^110^, ^122^ However, they preferred in‐person^78^, ^80^, ^91^ or blended learning^44^, ^100^, ^107^, ^112^, ^114^, ^117^, ^130^ over fully digital teaching. In digital settings, students appreciated the possibility of following the content of the lecture at any time and from anywhere.^65^, ^82^, ^83^ However, some challenges, such as reduced peer interactions and lack of motivation, were reported by students.^68^, ^78^, ^80^, ^129^

The use of WSIs and digital microscopy instead of glass slides and traditional microscopy gave better student performance in several studies,^46^, ^84^, ^126^, ^215^ and student acceptance rates indicated preference for digital microscopy.^37^, ^72^, ^75^, ^81^, ^125^ Some studies, however, found no significant difference in student performance when using digital microscopy compared with traditional microscopy,^108^, ^125^ and some students preferred traditional microscopy over digital microscopy.^46^, ^108^, ^125^ Educators and institutions, despite reporting technical challenges related to infrastructure such as internet connection and unstable technology,^103^, ^132^ appreciated the possibility of reaching out to a higher number of students in the digital microscopy courses as compared with being dependent on the infrastructure that comes with traditional microscopes.^83^, ^96^ With the integration of digital microscopy, access to microscopy courses is no longer limited by access to traditional microscopy and glass slide collections.

This section presents the comparison between traditional, digital, and blended learning methods in histology and pathology education. Digital microscopy was generally well‐received and helped expand access to courses, though some students and educators still faced technical challenges. Students appreciated the flexibility of digital learning but often preferred in person or blended formats due to better interaction and motivation. Consistent with broader literature in higher education, blended learning emerges as effective approach to overcome challenges posed by emerging technologies.^216^, ^217^, ^218^

### The accelerated use of digital microscopy during the COVID‐19 pandemic

A strong increase in the number of publications regarding the use of digital microscopy in histology and pathology teaching was seen during and following the COVID‐19 pandemic. The pandemic forced universities and institutions worldwide to adopt online teaching,^17^ accelerating the use of digital microscopy in histology and pathology education.^24^ Throughout the pandemic, educators were forced to find innovative approaches to teach and support student learning using various digital formats. Tools such as video conferencing, LMS, and presentation software played a crucial role in adapting teaching methods to digital environments.^100^, ^219^

During the COVID‐19 pandemic, asynchronous online methods were found beneficial for their adaptability,^51^ when students during the first phase of the pandemic were employed as auxiliary staff, and therefore, had to be able to attend classes more flexibly. However, students also reported trouble in separating study activities from home duties,^72^ found it harder to focus during online teaching compared with onsite teaching,^79^, ^96^, ^105^ and reported lower levels of confidence and engagement.^220^ After the pandemic, institutions and educators advocated the use of a blended approach, where digital resources made during the pandemic, were integrated into in‐person classes.^221^ The use of digital microscopy and digitized slides has persisted and became a central component of histopathological education in many institutions.^222^

### Future directions

Advancements in artificial intelligence (AI) can significantly influence the future of histology and pathology education, as adaptive learning has opened up the possibility of more personalized and data‐driven educational experiences.^38^, ^45^, ^61^, ^63^ Examples of AI‐based educational approaches are the use of learning analytics,^45^ decision tree algorithms to predict and support diagnoses,^38^ adaptive game‐like quizzes to develop “fast thinking” diagnostic skills,^63^ and data‐driven tracking of student performance.^61^ Adaptive learning can be beneficial for both students and educators, enabling a more efficient learning process based on data and providing practical solutions when access to pathologists is limited.^223^ However, while using AI brings many benefits, there are also challenges and ethical issues.^224^, ^225^, ^226^ Adaptive learning relies on information about the students, and by incorporating algorithms in digital learning tools, it is important to follow privacy laws like GDPR.^224^ Using AI technology as support in education can be of great value for both students and educators.^38^, ^45^, ^61^, ^63^ However, hands‐on practice, group discussions, and learning directly from educators are still essential elements for building a professional identity, and important medical skills, like critical thinking and decision‐making.

Simulation‐based tools, such as virtual reality (VR) and augmented reality, are potential new technologies in medical education. Thus far, they have been used predominantly for gross anatomy education, focusing on 3D anatomical visualization of structures that are much larger than the cellular and tissue‐level detail that is essential in histology and pathology teaching.^103^ One study described a VR environment to simulate a histology laboratory, allowing first‐year students to interact with light microscopes.^227^

Three‐dimensional (3D) digital microscopy can potentially make it easier for medical students to understand the organization of tissues in the body, spatial relationships, and how diseases affect and change tissue.^2^, ^228^ Some institutions have developed digital learning resources using 3D digital microscopy technology.^229^, ^230^, ^231^ However, managing 3D digital microscopy slides can be technically demanding and their value in histology and pathology education is uncertain.

Electron microscopy (EM) is a technology that produces micrographic images, enabling visualization of subcellular structures that are not visible through regular light microscopy. EM images are often included in histology books to illustrate cellular structures. Although rarely used, digital EM has been tested in histology and pathology education.^24^ Collections of digital EM images can be found in different repositories^232^, ^233^ and digital learning tools.^162^, ^174^ Its incorporation can offer new perspectives and help students identify subcellular structures.^234^

### Synthetizing findings relevant to the design and development of digital learning tools with digital microscopy integrated

This review identifies key functionalities in the design of a digital learning tool with digital microscopy integrated. These insights can guide the future design and development of such tools for histology and pathology education.

**FIGURE 4:**
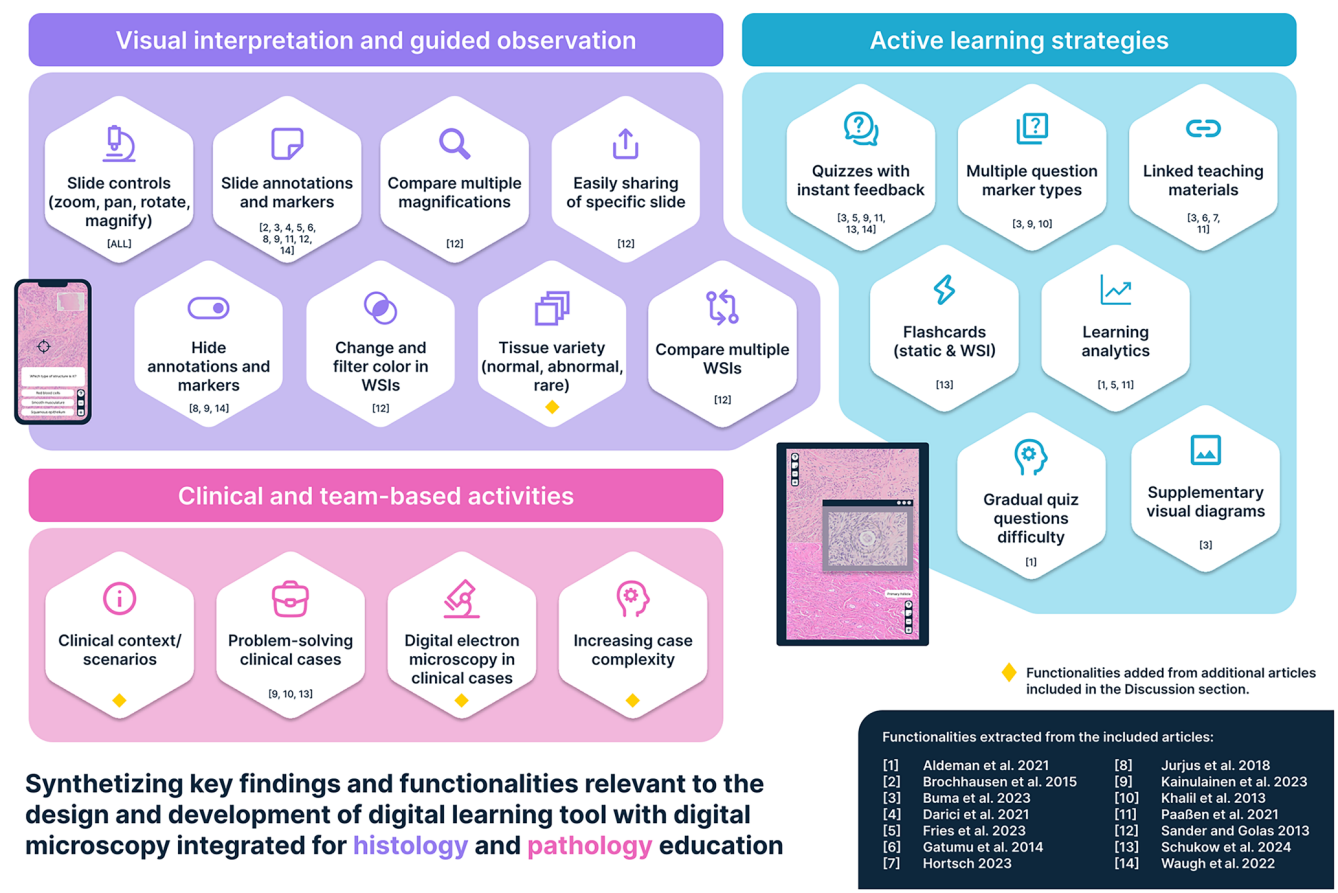
Synthesis of the key findings and functionalities relevant to the design and development of digital learning tools with digital microscopy integrated. These functionalities were identified from the data extracted from the included articles, reported in Table 5, and from additional articles that were included in the Discussion section.

Figure 4 synthesizes these key functionalities organized into three main categories that reflect the main findings related to teaching methods and assessment approaches.

### Limitations of the study

This study has limitations. Only studies published in English and from January 2013 were included. This may have excluded relevant research published in other languages and earlier work that can offer valuable insights into the development of early online teaching methods and educational practices. As the literature search focused on medical students and their educators, studies involving the use of digital microscopy for other histology or pathology learner groups were not included. Another limitation is the reliance on self‐reported data from students and educators, which may have introduced bias, as responses are influenced by personal perceptions and experiences. Furthermore, sources, such as theses, conference proceedings, book chapters, and reports were excluded from this review. However, relevant perspectives from such literature were included in the discussion. The study adhered to the journal's guidelines for systematic reviews, and articles that were not identified through the systematic literature search were not included in the results section. Several websites offering WSI collections and digital microscopy‐based digital learning tools were not identified by the search, since they had not been presented in published articles. Such websites can nevertheless be of interest to readers, and some have been included in the discussion.

## CONCLUSIONS

Histology and pathology education is evolving, driven by advancements in technology and the adoption of digital microscopy. The COVID‐19 pandemic accelerated a shift that was already underway, leading universities and institutions worldwide to rapidly adopt online teaching methods. In the post‐pandemic era, many universities and institutions have maintained and continued to adapt and improve digital and blended teaching. Institutions and educators need to stay abreast of developments in digital technologies to ensure relevant and adapted learning for medical students. This review maps the existing digital solutions found in contemporary literature and identifies opportunities for future development. Digital learning tools that integrate digital microscopy have gained attention and their use shape how histology and pathology are taught, assessed, and experienced. The use of such tools can foster active, collaborative, and independent learning, offering benefits while posing new challenges for educators and students. Further research should delve deeper into the interactions between students and educators, and pedagogical approaches that support digital and blended learning environments. This may facilitate the creation of dynamic learning environments that better prepare students for the challenges of modern medical practice.

## AUTHOR CONTRIBUTIONS

**Eleonora Nava:** Conceptualization; formal analysis; visualization; writing – original draft; writing – review and editing. **Ashis Jalote‐Parmar:** Conceptualization; formal analysis; visualization; writing – review and editing; supervision. **Cecilie Våpenstad:** Conceptualization; formal analysis; visualization; writing – review and editing; supervision; funding acquisition. **Marit Valla:** Conceptualization; formal analysis; visualization; writing – review and editing; supervision; funding acquisition; project administration.

## FUNDING INFORMATION

This work was supported by the Liaison Committee between the Central Norway Regional Health Authority and the NTNU Norwegian University of Science and Technology (project number 2022/787) and from the Norwegian Directorate for Higher Education and Skills (project number 21/04728‐442).

## APPENDIX A SCOPING REVIEW SEARCH STRATEGY

### A.1

**Database: PubMed**

Search date: May 24, 2024; updated on September 1, 2024.

**Table:**

#	Searches	Results
1	"Histology"[Mesh]	381,483
2	"Pathology"[Mesh]	50,142
3	histology[Title/Abstract]	163,296
4	pathology[Title/Abstract]	430,245
5	"microscopic anatomy"[Title/Abstract]	670
6	histopathology[Title/Abstract]	89,517
7	"virtual microscopy"[Title/Abstract]	461
8	#1 OR #2 OR #3 OR #4 OR #5 OR #6 OR #7	1,029,527
9	"Education, Distance"[Mesh]	7216
10	"digital learning"[Title/Abstract]	617
11	"online learning"[Title/Abstract]	5232
12	"e‐learning"[Title/Abstract]	5367
13	"hybrid learning"[Title/Abstract]	369
14	"blended learning"[Title/Abstract]	1820
15	#9 OR #10 OR #11 OR #12 OR #13 OR #14	17,239
16	#8 AND #15	308
17	#8 AND #15 Filters: **Full text, English, from 2013**	232

*Search string:* (("Histology"[Mesh]) OR ("Pathology"[Mesh]) OR (histology[Title/Abstract]) OR (pathology[Title/Abstract]) OR ("microscopic anatomy"[Title/Abstract]) OR (histopathology[Title/Abstract]) OR ("virtual microscopy"[Title/Abstract])) AND (("Education, Distance"[Mesh]) OR ("digital learning"[Title/Abstract]) OR ("online learning"[Title/Abstract]) OR ("e‐learning"[Title/Abstract]) OR ("hybrid learning"[Title/Abstract]) OR ("blended learning"[Title/Abstract])) Filters: Full text, English, from 2013

**Database: EMBASE**

Search date: 24th May 2024; updated on 1st September 2024.

**Table:**

#	Searches	Results
1	**'pathology'**/exp	1,949,389
2	**'histology'**/exp	1,664,551
3	histology:ti,ab,kw	255,633
4	pathology:ti,ab,kw	608,619
5	'microscopic anatomy':ti,ab,kw	797
6	histopathology:ti,ab,kw	137,373
7	'virtual microscopy':ti,ab,kw	686
8	#1 OR #2 OR #3 OR #4 OR #5 OR #6 OR #7	3,691,607
9	'distance learning'/exp	8859
10	'digital learning':ti,ab,kw	515
11	'online learning':ti,ab,kw	5142
12	'e‐learning':ti,ab,kw	7391
13	'hybrid learning':ti,ab,kw	363
14	'blended learning':ti,ab,kw	2066
15	#9 OR #10 OR #11 OR #12 OR #13 OR #14	19,018
16	#8 AND #15	495
17	#8 AND #15 AND (**'article'**/it OR **'article in press'**/it OR **'conference paper'**/it) AND [english]/lim AND [2013‐2024]/py	210

Search String: ('pathology'/exp OR 'histology'/exp OR histology:ti,ab,kw OR pathology:ti,ab,kw OR 'microscopic anatomy':ti,ab,kw OR histopathology:ti,ab,kw OR 'virtual microscopy':ti,ab,kw) AND ('distance learning'/exp OR 'digital learning':ti,ab,kw OR 'online learning':ti,ab,kw OR 'e‐learning':ti,ab,kw OR 'hybrid learning':ti,ab,kw OR 'blended learning':ti,ab,kw) AND ([article]/lim OR [article in press]/lim OR [conference paper]/lim) AND [english]/lim AND [2013‐2024]/py

**Database: ERIC ProQuest**

Search date: May 24, 2024; updated on September 1, 2024.

**Table:**

#	Searches	Results
1	MAINSUBJECT.EXACT.EXPLODE("Pathology")	6704
2	histology	166
3	pathology	5261
4	"microscopic anatomy"	14
5	histopathology	10
6	"virtual microscopy"	32
7	[S1] OR [S2] OR [S3] OR [S4] OR [S5] OR [S6]	8483
8	MAINSUBJECT.EXACT.EXPLODE("Distance Education")	27,437
9	MAINSUBJECT.EXACT.EXPLODE("Electronic Learning")	22,866
10	"digital learning"	1958
11	"online learning"	11,896
12	"e‐learning"	8786
13	"hybrid learning"	465
14	"blended learning"	8503
15	[S8] OR [S9] OR [S10] OR [S11] OR [S12] OR [S13] OR [S14]	57,791
16	[S7] AND [S15]	99
17	[S7] AND [S15] Filter: After 2013, English	67

**Database: Web of Science**

Search date: May 24, 2024; updated on September 1, 2024.

**Table:**

#	Searches	Results
1	**WC=(Pathology)**	762,525
2	**histology** (Topic)	162,227
3	pathology (Topic)	475,834
4	"microscopic anatomy" (Topic)	893
5	histopathology (Topic)	96,602
6	"virtual microscopy" (Topic)	794
7	#1 OR #2 OR #3 OR #4 OR #5 OR #6	1,409,829
8	"digital learning" (Topic)	3720
9	"online learning" (Topic)	29,172
10	"e‐learning" (Topic)	32,859
11	"hybrid learning" (Topic)	2594
12	"blended learning" (Topic)	9423
13	#8 OR #9 OR #10 OR #11 OR #12	70,389
14	#7 AND #13 AND Filter: After 2013, English, Article, Proceedings Paper	222

**Database: Scopus**

Search date: May 24, 2024; updated on September 1, 2024.

**Table:**

#	Searches	Results
1	TITLE‐ABS‐KEY (histology)	631,830
2	TITLE‐ABS‐KEY (pathology)	1,443,171
3	TITLE‐ABS‐KEY ("microscopic anatomy")	2071
4	TITLE‐ABS‐KEY (histopathology)	444,392
5	TITLE‐ABS‐KEY ("virtual microscopy")	408
6	#1 OR #2 OR #3 OR #4 OR #5	2,289,556
7	TITLE‐ABS‐KEY ("digital learning")	3774
8	TITLE‐ABS‐KEY ("online learning")	29,316
9	TITLE‐ABS‐KEY ("e‐learning")	89,510
10	TITLE‐ABS‐KEY ("hybrid learning")	2638
11	TITLE‐ABS‐KEY ("blended learning")	9674
12	#7 OR #8 OR #9 OR #10 OR #11	116,653
13	#6 AND #12 Filter: After 2013, English	750

Search String: ((TITLE‐ABS‐KEY (histology)) OR (TITLE‐ABS‐KEY (pathology)) OR (TITLE‐ABS‐KEY ("microscopic anatomy")) OR (TITLE‐ABS‐KEY (histopathology)) OR (TITLE‐ABS‐KEY ("virtual microscopy"))) AND ((TITLE‐ABS‐KEY ("digital learning") OR TITLE‐ABS‐KEY ("online learning") OR TITLE‐ABS‐KEY ("e‐learning") OR TITLE‐ABS‐KEY ("hybrid learning") OR TITLE‐ABS‐KEY ("blended learning"))) AND PUBYEAR > 2012 AND PUBYEAR < 2025 AND (LIMIT‐TO (LANGUAGE, "English"))
